# Comparative Evaluation of Crack Propagation During Root Canal Preparation in Primary Molar with Manual Instrumentation, ProTaper Universal and ProTaper Next Rotary File Systems: An *in vitro* Study

**DOI:** 10.30476/dentjods.2023.96668.1955

**Published:** 2024-12-01

**Authors:** Pooja Khakhar, Farhin Katge, Vamsi Krishna, Debapriya Pradhan, Shilpa Shetty, Devendra Patil

**Affiliations:** 1 Postgraduate Student, Dept. of Pediatric and Preventive Dentistry, Terna Dental College, Phase– II, Sector- 22, Nerul (west), Navi Mumbai, India; 2 Dept. of Pediatric and Preventive Dentistry, Terna Dental College, Phase – II, Sector- 22, Nerul (west), Navi Mumbai, India

**Keywords:** Molars, Dentin, Primary teeth, Root canal preparation

## Abstract

**Statement of the Problem:**

Root canal preparation with rotary files causes dentinal cracks in root canals of primary teeth affecting their longevity.

**Purpose:**

Nickel-titanium rotary files have been widely used for root canal preparation in primary teeth. The present study compared occurrence of dentinal microcracks in root canals of extracted primary molar teeth prepared using Hedstrom (H) files, ProTaper Universal rotary files, and ProTaper Next rotary file systems.

**Materials and Method:**

In this *in vitro* experimental study, 80 primary molar mesial root canals were randomly divided into four groups of 20 canals each (n=20). Group I was prepared with H files. Group II was prepared with ProTaper Universal rotary files using shaping files SX and S2. Group III was instrumented with ProTaper Next rotary files X1 and X2 while Group IV was left unprepared and served as control. Roots were stained with 1% methylene blue solution and sectioned perpendicular to the long axis at 2mm, 4mm, and 6mm from the apical foramen. Slices obtained were examined under the stereomicroscope. Data obtained were subjected to statistical analysis using chi square tests.

**Results:**

Dentinal microcracks were observed in groups prepared using H files, ProTaper Universal, and ProTaper Next rotary files. Highest percentage of cracked root canals (20%) was seen in Group I, prepared by H files. These cracks were complete in nature, found in apical sections and statistically significant (*p*= 0.016). Group III prepared with ProTaper Next showed 10% dentinal cracks, followed by ProTaper Universal group with 5% cracked root canals.

**Conclusion:**

H files may be considered more aggressive at apical third due to complete cracks produced.

## Introduction

The main aim of root canal instrumentation in primary teeth is to facilitate debris removal, disinfection, bacterial reduction, and obturation of the root canal system. The different methods of root canal preparation include manual techniques, automated systems, sonic, and ultrasonic instrumentation [ 1 - 2 ]. One frequent side effect of root canal preparation is root fracture, which typically results in tooth loss [ 3 ]. This is due to the fact that root canal preparation lead the dentin to become stressed, resulting in dentinal cracks that might enlarge into complete fractures when subjected to functional pressure [ 4 ].

The conventional technique of hand instrumentation using broaches and files is time consuming, which is sometimes complicated by problems of behaviour management in paediatric patients. When compared to conventional hand instrumentation, the introduction of nickel-titanium (NiTi) rotary instrumentation revolutionized root canal procedure by reducing operator fatigue, time needed for preparation, and procedural errors [ 5 ]. NiTi rotary files were first used for primary teeth's preparation for root canals by Barr *et al*. in 2000 [ 6 ]. The advantages of rotary files are numerous. Rotary files maintain the anatomy of primary teeth's curved root canals and ensure a funnel shaped preparation. They also shorten preparation time, enhancing cooperation of paediatric patients. However, rotary files may have the potential to cause complete and incomplete dentinal cracks in root canals [ 7 ]. The radicular dentin in primary teeth is thinner and the canals tortuous, thus raising concern of crack propagation in primary root canals with use of rotary file systems [ 8 ]. Shemesh *et al*. [ 9 ], 2009 first revealed occurrence of dentinal microcracks in radicular dentin of permanent teeth following instrumentation with rotary files. Various other studies have been conducted to assess dentinal microcracks following preparation with different rotary systems [ 10 - 11 ]. There is evidence that the Protaper Universal rotary system results in much more apical dentinal cracks in permanent teeth than the ProTaper Next rotary and WaveOne reciprocating systems [ 10 - 11 ]. Very few studies till date have been carried out in primary teeth [ 12 ]. Hence, there is a need to assess the occurrence of dentinal cracks during root canal preparation in primary teeth.

The aim of this *in vitro* study was to compare occurrence of dentinal microcracks in root canals of extracted primary molar teeth prepared using H files, ProTaper Universal rotary files, and ProTaper Next rotary file systems.

The null hypothesis for this study was that there is absence of dentinal microcracks in extracted primary molar root canal using manual instrumentation with H file, ProTaper Universal rotary file system, and ProTaper Next rotary file system. The alternative hypothesis for the same was that there was no difference in the occurrence of dentinal microcracks in root canals of extracted primary molar teeth instrumented with the above mentioned three techniques.

## Materials and Method

The study was carried out in the Department of Paediatric and Preventive Dentistry. The Institutional Review Board of Ethics granted approval for the study (Ethical clearance number: TDC/IRB-EC/127/2016).

### Selection of specimens

Extracted primary molar teeth were selected based on the inclusion and exclusion criteria. Primary maxillary and mandibular molar with mesial root with at least two-thirds of length present were included in the present study. Absence of external or internal pathological root resorption was another requirement for participation in the trial. Exclusion criteria were teeth with external cracks or defects and teeth exhibiting root caries. Selected teeth were maintained in Hanks balanced salt solution (Life Technologies Corporation, USA) until further use [ 13 ]. To rule out teeth with visible defects or cracks, the root surfaces of extracted teeth were examined at 10× magnification under a stereomicroscope (MOTIC SMZ-143 series). A double-sided diamond disc was then used to split teeth at the cemento-enamel junction (CEJ) while moving slowly and in cold water. It was followed by refining of the coronal access with a diamond round bur BR-46 (Mani Inc., Japan) and Gates Glidden drill, number 1 (Mani Inc., Japan). This was done to standardize the reference point while measuring the working length of primary molar root canals.

### Root canal procedure

The working length determination of root canals was done visually with inserting a 21mm, #10 K file (Dentsply Maillefer, Ballaigues, Switzerland) until it was just visible at the apical foramen. A silicone stopper was then adjusted at the CEJ and the file withdrawn. Working length was determined using an endoscale (Dentsply Maillefer, Ballaigues, Switzerland) by subtracting 1mm from this measurement [ 14 ]. Each canal was then negotiated with #15K file (Dentsply Maillefer, Ballaigues, Switzerland) till working length [ 13 ]. The root canals were then randomly divided by simple random sampling into four groups with 20 root canals in each group (n= 20). 

In Group I, root canals were prepared manually with H files (Dentsply Maillefer, Ballaigues, Switzerland).In Group II, root canals were prepared with ProTaper Universal (PTU) rotary nickel-titanium files (Dentsply Maillefer, Ballaigues, Switzerland). In Group III, root canals were prepared with ProTaper Next (PTN) rotary nickel-titanium files (Dentsply Maillefer, Ballaigues, Switzerland). In Group IV (Control group), canals were not prepared.

Single operator carried out all the root canal preparation procedure. Manual instrumentation in Group I was carried out using H files in a step back technique with an in and out filing motion. The root canals were prepared using 21 mm length files of size #15 to #30 with recapitulation and irrigation with 3% sodium hypochlorite solution [ 15 ].

In Group II, rotary instrumentation in crown down sequence was performed using a 21mm ProTaper Universal system (Dentsply Maillefer, Ballaigues, Switzerland). First, using a buccolingual brushing motion, shape file SX was placed in the canal 3mm beyond the root canal opening. This was followed by shaping file S2 till the working length of the canal in a brushing motion [ 16 ].

Instrumentation in Group III was performed with rotary ProTaper Next system (Dentsply Maillefer, Ballaigues, Switzerland). Crown-down technique was followed where X1 file was the first file used followed by X2 file till the working length of the root canal with an inward-outward brushing motion [ 17 ].

All NiTi rotary files were activated using an Endo-mate DT (NSK, Nakanishi, Japan) handpiece at speed between 250 and 350 rpm and a constant low torque between 2.5-3 Ncm as recommended by the manufacturer. The files were always examined for distortion or flute unwinding after being removed from the canal. The external root surface was kept moist throughout procedure with a gauze piece dipped in distilled water to prevent dehydration of the root. Each instrument (H file, ProTaper Universal and ProTaper Next rotary files) were used only in 5 canals which were later replaced with new files. A 2ml solution of 3% sodium hypochlorite (Parcan, Septodont, USA) was used to irrigate the root canals with 27 gauge needle after each instrument change and finally flushed with distilled water (Ranbaxy Laboratories, India). No instrumentation was done in root canals of Group IV and they served as control. 

### Staining and microscopic examination

After the root canal preparation procedure, all canals were dyed with 1% methylene blue solution (Pallav Chemicals & Solvents, India) [ 18 ]. Specimens were filled with the solution using a 30 gauge needle until working length was reached and the dye leaked through the apical foramen [ 15 ]. The dye in canal was then agitated with #15 reamer (Dentsply Maillefer, Ballaigues, Switzerland). Specimens were kept immersed in the dye solution for 24 hours in order to ensure complete staining. They were then washed in running water and root canals were irrigated with 5 ml of distilled water. The roots were then embedded in autopolymerizing epoxy resin (Dental Products of India, India) to prevent shrinking due to dehydration [ 13 ]. With a diamond disc operating slowly and water cooling, specimens were sectioned perpendicular to the long axis horizontally at 2mm, 4mm, and 6mm from the apical foramen in the coronal, middle, and apical third [ 11 ]. The slices obtained were polished with a 1200 grit waterproof abrasive paper for 60 seconds in order to reduce any scratches [ 13 ]. Sixty slices from each group were inspected for the existence of dentinal defects (microcracks) by 2 independent examiners who were blinded to the method of preparation of the root canals. Slices were examined under the stereomicroscope at 40× magnification. 

### Definition of defects

A crack is defined as a defect with complete crack lines extending from the inner root canal space up to the outer surface of the root. Incomplete crack lines are defects extending from the root canal space into the dentin without reaching the outer surface [ 19 ].

Roots were classified as cracked if at least 1 of the 3 sections obtained from each root showed either a complete or incomplete crack.

**Figure 1:**
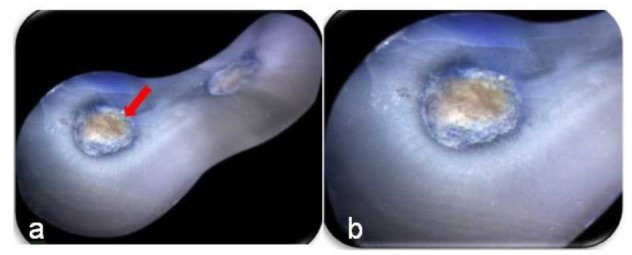
Specimen showing, **a:** Craze line that is unstained, extends from outer wall without connecting to canal lumen and not caused due to preparation, **b:** Magnified view

Craze lines were classified as all additional lines, including those that extended from the outer root surface into the dentin but did not enter the root canal space or any surface of the root canal wall (Figure 1).

### Statistical analysis

Data was subjected to analysis using windows based ‘MedCalc Statistical Software’ version 18.10 (MedCalc Software bvba, Ostend, Belgium; http://www.medcalc. org; 2018). Data for the cracks (complete and incomplete) were presented as numbers with percentages. Categorical data was compared between the groups using Chi-square tests at a significance level of *p*< 0.05. Agreement between the two examiners was analyzed using the Kappa’s test.

## Results

**Figure 2:**
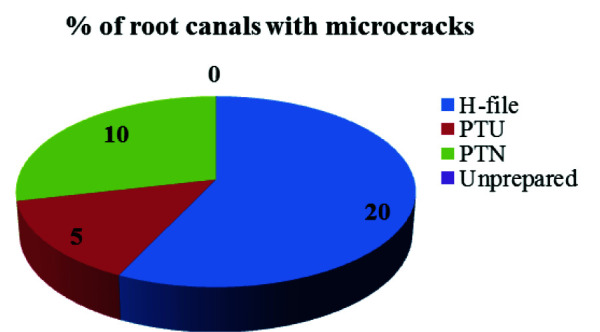
Percentage (%) of root canals presenting with dentinal microcracks in the four groups (H file – Hedstrom file, PTU - ProTaper Universal, PTN – ProTaper Next)

In present study, 80 root canals (mesial) were selected from extracted primary maxillary and mandibular molars (Figure 2).

### Detectable defects in the total initial sample

**Figure 3:**
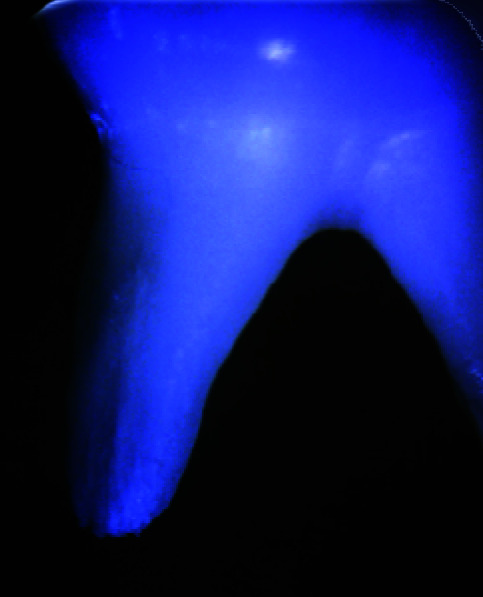
Pre-operative assessment of root surfaces under stereomicroscope at 10×

All the samples were screened preoperatively under the stereomicroscope at 10× magnification to exclude teeth with any external cracks or fractures. No such defects were found on examination (Figure 3).

### Incidence of dentinal microcracks after instrumentation

**Figure 4:**
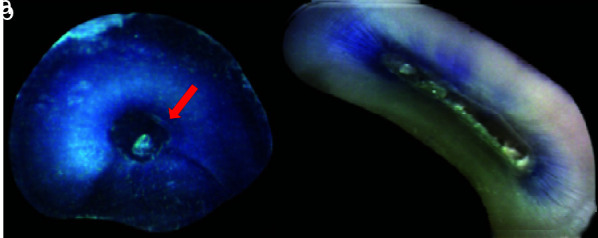
Specimens of Group I showing, **a:** Complete crack in apical section, **b:** No crack

**Figure 5:**
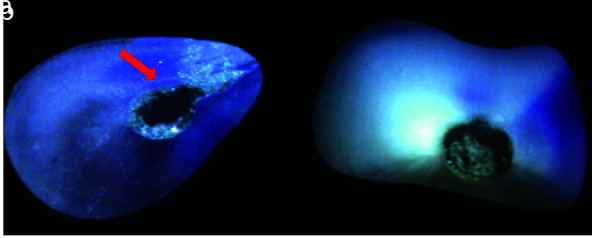
Specimens of Group II showing, **a:** Incomplete crack in coronal section, **b:** No crack

**Figure 6:**
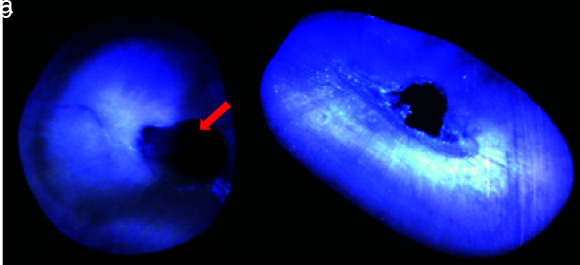
Specimens in Group III showing, **a:** Incomplete crack in coronal section, **b:** No crack

**Figure 7:**
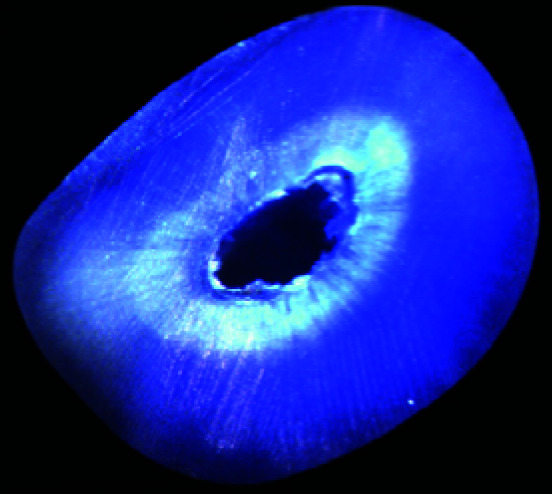
Specimen in Group IV showing no crack

In Group I after instrumentation with H files, 4 out of 20 (20%) root canals presented with dentinal microcracks (Table 1). This was statistically significant (*p*= 0.016) (Figure 4). In Group II prepared with ProTaper Universal NiTi rotary files, 1 out of 20 (5%) root canals presented with dentinal microcracks (Figure 5). In Group III prepared with ProTaper Next NiTi rotary files, 2 out of 20 (10%) root canals showed dentinal microcracks (Figure 6). In Group IV that was unprepared, no cracks were detected (Figure 7).

**Table 1:** Percentage and number of root canals presenting with dentinal microcracks in the four groups (H file- Hed-strom file, PTU- ProTaper Universal, PTN- ProTaper Next)

Microcracks present	Groups			
	H file	PTU	PTN	Unprepared
Percentage (%) of root canals within Group	20%	5%	10%	0
Number of root canals	4	1	2	0

### Location and Type of microcracks

### Coronal third

Cracks were absent in the coronal third in the group prepared with H files. One (5%) root canal showed incomplete crack in Group II prepared by ProTaper Universal rotary files while 2 (10%) root canals showed incomplete cracks in Group III instrumented using ProTaper Next rotary files. This was statistically not significant (*p*= 0.283) (Table 2).

**Table 2:** Percentage and number of root canals presenting with dentinal microcracks in the four groups in the coronal third, middle third and apical third (H file- Hedstrom file, PTU- ProTaper Universal, PTN- ProTaper Next)

Location	Crack propagation	Groups				Chi-Square value	p Value
		H file	PTU	PTN	Unprepared		
Coronal third	Incomplete crack	0	5% (1)	10% (2)	0	3.810a	.283
	Complete crack	0	0	0	0		
Middle third	Incomplete crack	0	0	0	0	-	-
	Complete crack	0	0	0	0		
Apical third	Incomplete crack	0	0	5% (1)	0	15.573a	.016*
	Complete crack	20% (4)	0	0	0		

* *p*< 0.05 indicates a statistically significant value

### Middle third

No cracks were seen in any of the groups in the middle third (Table 2).

### Apical third

In Group I prepared with H files, 4 (20%) root canals presented with complete cracks in the apical third. This was statistically significant (*p*= 0.016). ProTaper Universal group did not show any cracks in apical third while 1 (5%) root canal of ProTaper Next group showed an incomplete crack in the apical third (*p*= 0.016) (Table 2).

The value for the measure of agreement between the two examiners assessed by Kappa statistics was found to be 0.831 (*p*= 0.0001). This denoted very good agreement between the examiners.

## Discussion

The removal of dentin during root canal preparation either by manual or rotary instrumentation causes stress and strain that can induce defects or cracks in the dentinal wall [ 20 ]. Such cracks on gradual propagation over a period of time may lead to fracture of the root of endodontically treated teeth [ 21 ]. Vertical root fracture of such teeth due to these stresses is one of the undesirable complications that affects the long-term retention and longevity of such teeth. This assumes greater importance in primary dentition as early extraction due to such failures can have an adverse impact on the developing occlusion and dentofacial skeleton of the child. 

In the present study, Gates Glidden drill was used for coronal access refining. The diameter of Gates Glidden drill (size 2) is usually considered safe for the cervical preflaring in root canal preparation, however use of bigger diameters drills can cause inadvertent dentin removal [ 22 ]. Hence, smaller diameter with size 1 of Gates Glidden drill was used in the present study. Additionally, Akhlaghi *et al*. [ 23 ] demonstrated that Gates Glidden drills used in any order are appropriate, secure, and economical for preflaring of mandibular first molar root canals while maintaining the root thickness of furcation zones. 

There are various factors, which may lead to dentinal cracks during root canal instrumentation. Instrument related factors include file taper (constant or progressive), size, cross-sectional geometry, tip design, and flute form [ 24 ]. Arslan *et al*. [ 7 ] demonstrated that the wider the taper, the more root dentin is removed and greater the chance of root fracture. The kinematics of movement (rotating or reciprocating) of the NiTi files is another influencing factor [ 13 ]. Reciprocal motion is comprised of repeated clockwise (releasing) and counter clockwise (cutting) rotation. This permits the file to be continuously released as it is inserted into the dentin, hence lowering the torsional and flexural stresses. This may contribute in decreasing the incidence of dentinal damage. However, other studies suggest that reciprocating devices may be more likely to result in the development and spread of dentinal microcracks [ 25 ]. This is due to the fact that traditional preparation often results in fewer dentinal defects than root canal preparation incorporating a single large-tapered reciprocating instrument, which quickly removes a significant amount of dentin. Furthermore, reciprocal motion appears to facilitate the movement of debris towards the apex, which might increase torsional stresses [ 26 ]. Hence, in the present study, both the rotary file systems used were of continuous rotation. Microcracks may also be found in intact extracted teeth and these could be a confounding variable in the interpretation of results. Thus, before commencement of this study, extracted teeth were assessed preoperatively under the stereomicroscope to exclude teeth with any such detectable external cracks.

Besides instrument design, sectioning of the roots could also induce dentinal damage. Before sectioning the roots in this investigation, a dyeing technique was used. As a result, any fractures created by sectioning processes during or after did not absorb dye and were not stained [ 18 ].

In this study, there was absence of dentinal cracks in the negative control (unprepared) group, which was in accordance to the study by Yoldas *et al* [ 27 ]. Optical coherence tomography and computed microtomography are some of the other methods for the inspection of dentinal cracks [ 20 , 28 ] that do not require root sectioning for crack examination. However, divergent results were observed for the above methods. Thus, stereomicroscopy, which is one of the most common methods of assessment, was used in our study. Özyürek *et al*. [ 12 ] compared dentinal cracks in primary teeth caused by Reciproc, WaveOne Gold, and ProTaper Next NiTi rotary files. Root canals were prepared using RPC R25 (25/.08), WaveOne Gold Primary (25/.07) file and ProTaper next X1 (17/.04) and X2 (25/.06) respectively in the three groups. Dentinal cracks were found in all three file groups. In terms of the observed total dentin fracture, the WaveOne Gold and ProTaper Next groups had significantly less dentinal cracks than the Reciproc group. In comparison to the WaveOne Gold and ProTaper Next groups, the Reciproc group had more dentinal fractures in the apical area. Our study's findings show that all three tested groups of H files, ProTaper Universal, and ProTaper Next files had dentinal cracks. Interestingly, in contrast to the study by Özyürek *et al*. [ 12 ], highest percentage of cracked root canals (20%) were observed in the H file group followed by ProTaper Next group with 10% cracked root canals while only 5% root canals in ProTaper Universal group were cracked. 

In our study, complete cracks were seen only in the H file group in the apical section. The root canals in this group were instrumented till file size #30 (.02), which has an apical diameter of 0.30 mm. This apical tip diameter is greater than the tip diameter of the last file used at working length of the other two rotary file systems (S2=0.20mm and X2= 0.25mm). This may have contributed to increased stress and hence the complete crack seen in the H file group. Dentinal microcracks, found in the other two groups prepared with ProTaper Universal and ProTaper Next, were incomplete in nature and did not extend to the outer dentinal wall. 

According to results of present study, in the group prepared with H files, 20% of the root canals showed cracks in the apical section and it was statistically significant (*p*= 0.016). No cracks in this group were detected in the coronal and middle sections. This may be attributed to the increased strain in the apical third due to larger tip diameter (0.30 mm) of H files used at working length. In the group prepared with ProTaper Universal file system, 5% of root canals showed cracks in the coronal section with no cracks in apical and middle sections. In the ProTaper Next group, 10% canals presented with dentinal microcracks, which comprised of two coronal sections and one apical section. The cracks produced in the apical section by ProTaper Next rotary files were statistically significant (*p*= 0.016). Studies carried out in permanent teeth showed ProTaper Universal files to cause significantly more dentinal cracks at the apical third than ProTaper Next rotary files [ 10 - 11 ]. These findings are in contrast to results of our study in primary teeth that showed statistically significant cracks in apical third by ProTaper Next rotary files. This difference may be attributed to the fact that primary teeth begin to show physiologic root resorption that causes thinning of apical root dentin. Also, the increased taper (6%) and larger apical tip diameter of ProTaper Next file (X2 = 0.25 mm) compared to ProTaper Universal files may have led to statistically significant crack in the apical third in ProTaper Next group compared to ProTaper Universal group. Also, in the study conducted by Özyürek *et al*. [ 12 ], the highest percentage of tooth slices with defects in the ProTaper Next group were found in the middle third (40%) followed by coronal third (35%) and lastly apical third (20%). While in our study, highest cracks in ProTaper Next group were in the coronal third followed by apical third. This difference in occurrence of cracks in ProTaper Next group in the present study and that by Özyürek *et al*. [ 12 ] may be attributed to the differences in the scoring criteria used for cracks.

Certain other factors such as the storage of teeth after extraction and environmental conditions may affect the occurrence of cracks. In the present study, roots were embedded in acrylic resin without simulation of the periodontal ligament. This may be considered one of the limitations of the study. Also higher resolution techniques such as computed microtomography or infrared thermography can be used for assessment of pre-existing cracks, as certain cracks may not have been detected by stereomicroscopy in this study. Use of these techniques may have also avoided the procedure of sectioning of root into slices. Besides these, masticatory load on the teeth and parafunctional habits are some of the other factors affecting crack formation in the root canals, which could not be analyzed in this *in vitro* study.

## Conclusion

Within the limitations of this *in vitro* study, dentinal microcracks occurred in root canals following preparation with all three file systems, Hedstrom files, ProTaper Universal, and ProTaper Next rotary files. The percentage of cracked root canals found was highest in the group prepared with H files followed by ProTaper Next rotary files and ProTaper Universal rotary files. Complete cracks were observed with Hedstrom files, in the apical third whereas incomplete cracks were found in the ProTaper Universal rotary files and ProTaper Next rotary files.

## References

[1] Aminabadi NA, Asl Aminabadi N, Jamali Z, Shirazi S ( 2020). Primary tooth pulpectomy overfilling by different placement techniques: A systematic review and meta-analysis. J Dent Res Dent Clin Dent Prospects.

[2] ( 2016). Guideline on pulp therapy for primary and immature permanent teeth. Pediatr Dent.

[3] Mizuhashi F, Watarai Y, Ogura I ( 2022). Diagnosis of Vertical Root Fractures in Endodontically Treated Teeth by Cone-Beam Computed Tomography. J Imaging.

[4] Das S, Pradhan PK, Lata S, Sinha SP ( 2018). Comparative evaluation of dentinal crack formation after root canal preparation using ProTaper Next, OneShape and Hyflex EDM. J Conserv Dent.

[5] Mohamed RH, Abdelrahman AM, Sharaf AA ( 2022). Evaluation of rotary file system (Kedo-S-Square) in root canal preparation of primary anterior teeth using cone beam computed tomography (CBCT)-in vitro study. BMC Oral Health.

[6] Barr ES, Kleier DJ, Barr NV ( 2000). Use of nickel-titanium rotary files for root canal preparation in primary teeth. Pediatr Dent.

[7] Arslan H, Karataş E, Capar ID, Ozsu D, Doğanay E ( 2014). Effect of ProTaper Universal, Endoflare, Revo-S, HyFlex coronal flaring instruments, and Gates Glidden drills on crack formation. J Endod.

[8] Panda A, Shah K, Budakoti V, Dere K, Virda M, Jani J ( 2021). Evaluation of microcrack formation during root canal preparation using hand, rotary files and self-adjusting file in primary teeth: An in vitro study. J Dent Res Dent Clin Dent Prospects.

[9] ( 2009). The effects of canal preparation and filling on the incidence of dentinal defects. Int Endod J.

[10] Karataş E, Gündüz HA, Kırıcı DÖ, Arslan H, Topçu MÇ, Yeter KY ( 2015). Dentinal crack formation during root canal preparations by the twisted file adaptive, ProTaper Next, ProTaper Universal and WaveOne instruments. J Endod.

[11] Capar ID, Arslan H, Akcay M, Uysal B ( 2014). Effects of ProTaper Universal, ProTaper Next and HyFlex instruments on crack formation in dentin. J Endod.

[12] Özyürek T, Uslu G, Yılmaz K ( 2017). Effect of different nickel-titanium rotary files on dentinal crack formation during root canal preparation in primary molars: a laboratory study. Turk Endod J.

[13] Li SH, Lu Y, Song D, Zhou X, Zheng QH, Gao Y, et al ( 2015). Occurrence of dentinal microcracks in severely curved root canals with ProTaper Universal, WaveOne and Pro-Taper Next File systems. J Endod.

[14] Kfir A, Elkes D, Pawar A, Weissman A, Tsesis I ( 2017). Incidence of microcracks in maxillary first premolars after instrumentation with three different mechanized file systems: a comparative ex vivo study. Clin Oral Investig.

[15] Katge F, Chimata VK, Poojari M, Shetty S, Rusawat B ( 2016). Comparison of cleaning efficacy and Instrumentation time between Rotary and manual instrumentation techniques in primary teeth: an in vitro study. Int J Clin Pediatr Dent.

[16] Kalita S, Agarwal N, Jabin Z, Anand A ( 2021). Comparative evaluation of cleaning capacity and efficiency of Kedo-S pediatric rotary files, rotary ProTaper, and Hand K files in primary molar pulpectomy. Int J Clin Pediatr Dent.

[17] Rao A A, Pandya D, Roy S, Upadhyay K, Gupta S, Pal A ( 2018). Comparison of instrumentation time and cleaning efficacy of manual k-file, rotary protaper universal and rotary protaper next in primary anterior teeth: an in vitro study. Int J Sci Res (Ahmedabad)..

[18] Cassimiro M, Romeiro K, Gominho L, De Almeida A, Silva L, Albuquerque D ( 2018). Effects of Reciproc, ProTaper Next and WaveOne Gold on Root Canal Walls: A Stereomicroscope Analysis. Iran Endod J.

[19] Tejaswi S, Singh A, Manglekar S, Ambikathanaya UK, Shetty S ( 2022). Evaluation of dentinal crack propagation, amount of gutta percha remaining and time required during removal of gutta percha using two different rotary instruments and hand instruments: an in vitro study. Niger J Clin Pract.

[20] Helvacioglu-Yigit D, Aydemir S, Yilmaz A ( 2015). Evaluation of dentinal defect formation after root canal preparation with two reciprocating systems and hand instruments: an in vitro study. Biotechnol Biotechnol Equip.

[21] Shemesh H, van Soest G, Wu MK, Wesselink PR ( 2008). Diagnosis of vertical root fractures with optical coherence tomography. J Endod.

[22] Sousa K, Andrade-Junior CV, Silva JM, Duarte MA, De-Deus G, Silva EJ ( 2015). Comparison of the effects of TripleGates and Gates-Glidden burs on cervical dentin thickness and root canal area by using cone beam computed tomography. J Appl Oral Sci.

[23] Akhlaghi NM, Naghdi A, Bajgiran LM, Behrooz E ( 2014). Computed tomography evaluation of residual root thickness after pre-flaring using gates Glidden drills: The sequence effect. J Conserv Dent.

[24] Ustun Y, Aslan T, Sagsen B, Kesim B ( 2015). The effects of different nickel-titanium instruments on dentinal microcrack formations during root canal preparation. Eur J Dent.

[25] Bürklein S, Tsotsis P, Schäfer E ( 2013). Incidence of dentinal defects after root canal preparation: reciprocating versus rotary instrumentation. J Endod.

[26] Bürklein S, Schäfer E ( 2012). Apically extruded debris with reciprocating single-file and full-sequence rotary instrumentation systems. J Endod.

[27] Yoldas O, Yilmaz S, Atakan G, Kuden C, Kasan Z ( 2012). Dentinal microcrack formation during root canal preparations by different NiTi rotary instruments and the self-adjusting file. J Endod.

[28] De-Deus G, Silva EJ, Marins J, Souza E, de Almeida Neves A, Belladonna FG, et al ( 2014). Lack of causal relationship between dentinal microcracks and root canal preparation with reciprocation systems. J Endod.

